# Doctors’ learning experiences in end-of-life care – a focus group study from nursing homes

**DOI:** 10.1186/s12909-017-0865-8

**Published:** 2017-01-31

**Authors:** Anette Fosse, Sabine Ruths, Kirsti Malterud, Margrethe Aase Schaufel

**Affiliations:** 1 Research Unit for General Practice, Uni Research Health, Bergen, Norway; 2 Department of Global Public Health and Primary Care, University of Bergen, Bergen, Norway; 3 Department of Public Health, The Research Unit for General Practice and Section of General Practice, University of Copenhagen, Copenhagen, Denmark; 4 Department of Thoracic Medicine, Haukeland University Hospital, Bergen, Norway

**Keywords:** Medical education, Qualitative research, Focus group, Doctor-/patient relationship, Internship and residency, Professional development, Nursing home, End of life care, Death

## Abstract

**Background:**

Doctors often find dialogues about death difficult. In Norway, 45% of deaths take place in nursing homes. Newly qualified medical doctors serve as house officers in nursing homes during internship. Little is known about how nursing homes can become useful sites for learning about end-of-life care. The aim of this study was to explore newly qualified doctors’ learning experiences with end-of-life care in nursing homes, especially focusing on dialogues about death.

**Methods:**

House officers in nursing homes (*n* = 16) participated in three focus group interviews. Interviews were audiotaped and transcribed verbatim. Data were analysed with systematic text condensation. Lave & Wenger’s theory about situated learning was used to support interpretations, focusing on how the newly qualified doctors gained knowledge of end-of-life care through participation in the nursing home’s community of practice.

**Results:**

Newly qualified doctors explained how nursing home staff’s attitudes taught them how calmness and acceptance could be more appropriate than heroic action when death was imminent. Shifting focus from disease treatment to symptom relief was demanding, yet participants comprehended situations where death could even be welcomed. Through challenging dialogues dealing with family members’ hope and trust, they learnt how to adjust words and decisions according to family and patient’s life story. Interdisciplinary role models helped them balance uncertainty and competence in the intermediate position of being in charge while also needing surveillance.

**Conclusions:**

There is a considerable potential for training doctors in EOL care in nursing homes, which can be developed and integrated in medical education. This practice based learning arena offers newly qualified doctors close interaction with patients, relatives and nurses, teaching them to perform difficult dialogues, individualize medical decisions and balance their professional role in an interdisciplinary setting.

## Background

The transition from being a student to becoming a doctor is crucial for newly qualified doctors. Internship is an important period during this process. Experiences during internship vary from valuable to nearly unbearable depending on the content, structure and supervision [1, 2]. Clinical training during medical school is mostly performed in hospitals, focusing on diagnostics and curative treatment. Newly qualified doctors receive little formal training in end-of-life (EOL) care in hospitals and are not prepared to meet challenges regarding death and dying [3, 4].

In Norway, serving as house officers in nursing homes during primary care internship is mandatory, but organisation and content vary considerably. In nursing homes, newly qualified medial doctors meet frail old people living with death in sight, making this arena relevant for developing skills and reflections about EOL care [5]. Nursing homes are common places for spending the last period of life in many high-income countries. Providing EOL care is therefore one of the main tasks for health personnel in nursing homes. Organisation and services provided in nursing homes vary between countries [6]. In Norway, the dominant model of nursing home care is a nursing model with a registered nurse (e.g. nurse director) with administrative responsibilities who oversees care of residents working with a staff team. Medical care is provided by part-time employed GPs (60–65%), other specialists (e.g. geriatrics, internal medicine), or full time employed nursing home doctors [7]. Patients and their relatives request doctors and nurses who are confident in EOL care and in communication about death and dying [8], but health care professionals often find dialogues about death difficult [9]. Doctors learn to provide palliative care mostly through learning by doing [10]. Nursing homes can provide students and newly graduated doctors and nurses with teamwork competence through intra- and inter-professional learning [11–13]. However, these institutions are usually not utilized purposively as learning arenas [14].

As medical doctors with long experience from general practice (AF, SR, KM), nursing home medicine (AF, SR) and hospital palliative care (MAS), we share a concern regarding the quality of EOL care. Furthermore, we shared the preconception that medical education, including the maturation and development of new doctors through internship, is a key factor ameliorating this important field. From this perspective, we set up a study exploring how newly qualified doctors’ clinical experiences from nursing homes may provide access to learning about death and dying, especially focusing on dialogues about death.

### Theoretical perspectives

We were inspired by Lave and Wenger’s theory about *situated learning* and *legitimate peripheral participation* [15]. According to this theory, learning is not only the reception of factual knowledge or information, but perhaps even more a process situated within activity, context and culture. The learning process develops through participation in communities of practice, starting in the periphery, and gradually moving towards the center as skills, knowledge and culture is internalized by the learner. These theoretical perspectives explain how apprentices’ learning experiences from a practice environment little by little contribute to the development from being novices in their field into skilled masters. During internship, doctors experience a process of transformation from medical students to responsible professionals. This happens gradually as they adapt to culture and activity on the hospital ward, in general practice or the nursing home in which they practice. We used Lave and Wenger’s theory to shed light on how newly qualified doctors learn about death and dying in the specific context of nursing homes.

## Methods

### Study setting and design

Nursing homes provide twenty-four hours care and medical treatment to heterogeneous populations of frail, old people during long-term or short-term stays. About 45% of deaths in Norway take place in these facilities [16]. Nearly 80% of the patients suffer from dementia [17], which can be a challenge to communication, especially dialogues about difficult decisions [18].

We conducted a qualitative study based on three focus group interviews, each with 3–8 participants, carried out in 2014–2015. During primary care internship, interns attended three coaching assemblies. The focus group interviews took place at the last assembly, when the doctors had experienced dialogues about death and EOL care in nursing homes.

### Participants

We recruited a purposive sample of 16 newly qualified doctors during their internship in nursing homes in one county in Norway, using preexisting coaching groups and e-mail invitations. We aimed for variation in gender (12 women, 4 men), age (26–37 years), background, place of graduation and internship location. Participants’ nursing home practice varied from a few days of observation to independent work as doctors responsible for medical services, with variable access to supervisors. The researchers did not know any of the participants from before. Participants knew each other from the coaching groups.

### Data collection

Each focus group interview lasted approximately 90 min and was conducted according to established principles [19, 20]. Based on an interview guide, the moderator (AF) invited the participants to share good and bad experiences with EOL care in nursing homes. They were also encouraged to tell about preparatory dialogues with patients and relatives and reflect on how experiences with death and dying in nursing homes influenced their view on EOL care and doctor’s role in this regard. We asked for specific learning experiences in EOL care illuminating this process. Our focus was how communities of practice inspired and contributed to personal development and forming of doctor’s role. The last author (MAS) took field notes. After three focus group interviews, the research group assessed the material rich enough to shed light on our research question. The interviews were audiotaped and transcribed verbatim by the first author and moderator (AF).

### Analysis

**Fig. 1:**
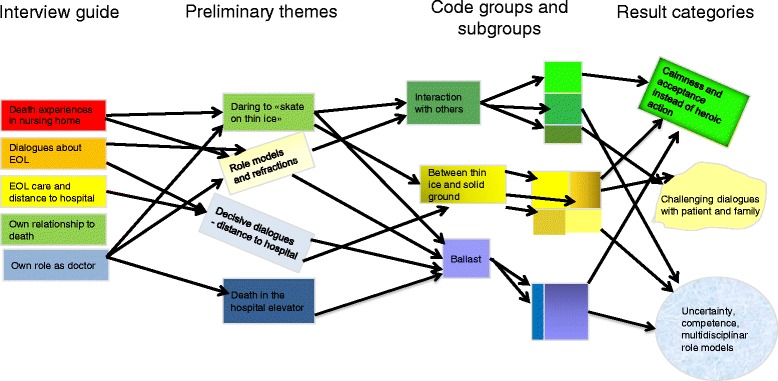
Analytic process, modified with permission after Malterud 2011 [21]

Data analysis was performed according to systematic text condensation through the following steps: 1) Reading the material to obtain an overall impression, bracketing preconceptions, 2) identifying units of meaning, representing different aspects of the participants’ learning experiences and coding for these, 3) condensing and abstracting the meaning within each of the coded groups, and 4) summarizing the contents of each coded group to generalized descriptions and concepts reflecting participants’ most prominent learning experiences, Fig. 1 [21]. The figure illustrates keywords during the development of categories, as well as the non-linearity of the analytic process. Codes are malleable and vary in the course of analysis. Analysis was performed stepwise, with new focus group interviews supplementing the sample. Categories and findings were developed from the empirical data using editing analysis style [22]. Analysis was supported by Lave and Wenger’s theory regarding situated learning [15]. All authors were involved during the analysis process. We focused especially on the participants’ experiences regarding dialogues with patients, relatives and nurses, and decision-making processes concerning EOL care in nursing homes.

## Results

Newly qualified doctors explained how nursing home staff’s attitudes taught them how calmness and acceptance could be more appropriate than heroic action when death was imminent. Shifting focus from disease treatment to symptom relief was demanding, yet participants comprehended situations where death could even be welcomed. Through challenging dialogues dealing with family members’ hope and trust, they learnt how to adjust words and decisions according to family and patient’s life story. Interdisciplinary role models helped them balance uncertainty and competence in the intermediate position of being in charge while also needing surveillance. Below, we elaborate these findings. Quotations illustrating the findings are assigned pseudonyms.

### Calmness and acceptance can be more appropriate than heroic action when death is imminent

Participants perceived the calm and accepting attitudes of nursing home staff when patients approached the end of life as a sharp contrast to the therapeutic efforts and stress associated with death and dying in hospital. Earlier during internship, several participants had encountered disturbing events with nursing home patients who were admitted to hospital; for example a patient dying in the elevator during transportation. One participant had witnessed how patients who died in the hospital immediately were transported to the cooling facility because the room should be prepared for another patient. This differed from rituals unfolding when a patient died in the nursing home, where nothing disturbed the deceased staying in their beds for a while, a candle being lit and family gathering there. In the nursing home, the participants learnt that their mere presence could provide more support to patient, family and nurses than frantic activity. A young doctor told about her first experience with death in the nursing home, when she was called upon to see a very old woman with dementia who was dying:

*“I didn’t know what to do. The patient was not conscious; it was not possible to do a lot of bedside examinations. So I just tried to make some contact with her and checked heart and lungs without really knowing how this could be useful. And then I understood that perhaps this was sufficient, that the doctor had been there, standing by.” (Karen)*

Participants found it to be demanding when shifting focus from treating diseases to symptom relief and quality of life. They experienced uncertainty in decision-making processes, for example whether they should admit a patient to hospital or not when health deteriorated. Patients and families often denied hospital admission, fearing stressful transportation and a long stay in the emergency room. Participants learnt that palliative care provided in the nursing home could be a better alternative than hospital admission.

The young doctors explained how engaging in patients’ lives and treatment could lead to emotional affection. One participant told about grief when a nursing home patient, whom she had got acquainted with during her internship, died abruptly. On the other hand, she also experienced relief and acceptance because the old woman and her family were well prepared. Participants learnt how they could contribute to making a deathbed peaceful and dignified together with the nurses, and that approaching death even could be positive and welcomed. A young doctor who had followed several EOL trajectories in the nursing home, described her feelings like this:

*“I think it was kind of a good feeling when the patient refused to eat and to take medication, and the family agreed that time had come for letting go. I could give palliative treatment, and the situation felt quite good, actually. I find it soothing that death can be something good, compared to how death was perceived in hospital – if I can put it that way?” (Judy)*

### Challenging and close relationships with patient and family may enrich end-of-life dialogues

Participants often considered their relationship with patients’ families differently in nursing homes compared to hospital. They experienced how long and often repeated conversations with patient and family provided insight into the patient’s life story. The nursing home setting offered the possibility of getting to know patients and families over a time span longer than most hospital stays. In this setting it fell more natural to discuss prognosis, treatment and the patient’s and family’s expectations and wishes. Some participants elaborated on powerful experiences with spouses following their beloved ones very closely, not wanting to lose hope. Sometimes such relatives were regarded as “difficult” by the nursing staff. However, when the interns perceived how a long life together promoted the wish for doing all the best for their ill partner, they comprehended their behavior. This often made communication and decision-making easier. Another tough situation described was disagreement between family members concerning the patients’ condition. A male doctor experienced how he had to perform flexible judgement:

*“…the most taxing is to handle the dynamics of the family. Liquid infusion should probably have been discontinued earlier, but I had to compromise in order to give the family some time to come to terms with it. (Rick)*

Even if it could be difficult coping with relatives who expressed disagreement and distrust concerning prognosis and treatment, the interns in retrospect considered such experiences as valuable. Through challenging dialogues where they had to balance realistic prognosis against family members’ hope and trust, participants learnt how to gently adjust words and information according to their knowledge of the family and patient’s life story to make the conversation more bearable for both sides. A female doctor who had conducted several dialogues with patients and families, shared her experiences:

*“Every meeting and dialogue with the patients’ families is pure gold, even though some of them have been difficult. I bring the good and the bad experiences with me, and learn from them for my future work” (Lisa)*

Participants explained how provision of information was an important medical task towards end of life, although it could be demanding. Some young doctors imparted how they talked openly with patients with life-threatening illness and their family, even if more experienced doctors were reluctant to do so. As they sat down and let patients and relatives ask questions, they became aware that they as doctors possessed knowledge that patients and families needed to clarify important matters. Through this process, some of the participants experienced that their role as doctors became easier. One participant described how combining comprehensive information about poor prognosis and available treatment choices with the exposure of her own uncertainty and ambivalence in decision-making could build trust. Another participant reported from a dialogue she had had with an old woman with cancer and her son:

*“It wasn’t as hard as I’d thought. I think that we all were aware of the advanced cancer and that she probably soon would die from it. So for me it was a positive experience.”(Mary)*

### Balancing uncertainty and competence inspired by multidisciplinary role models

Participants from different nursing homes reported quite different internship organisation. Some had been working very independently, having full responsibility, while others only had been observers for a couple of days. The latter expressed frustration, and would have preferred more nursing home practice and responsibility in order to learn more. Participants also described a great variety regarding nursing home doctors as role models. Some supervisors demonstrated good communication with patients and families, while others avoided confronting conversations. Participants sometimes disagreed with their supervisor, but found it difficult to oppose the older doctor who had extensive clinical experience and knowledge. Good as well as bad role models gave the participants useful reflection upon their own development into the doctor’s role. Observation of a skilled nursing home doctor relating to patient and family offered an inspiring example of how a dialogue about end of life could be performed, outlined by one of the interns like this:

*“While listening, I thought about how I should have performed this conversation. And I found that the way she did it was very good, and that this is a way for me to carry out such a dialogue” (Sandy)*

When participants were to make difficult decisions or perform challenging dialogues, they often picked up useful information from experienced nurses who knew patient and family. On the other hand, some nurses were insecure and demanded examinations or admittance to hospital even if the doctor found it unnecessary. In such situations, participants were stumped on how to collaborate with the staff. They acknowledged nurses and nurse assistants as the ones who knew the patients best and often had long experience, while at the same time they would sometimes call for the doctor on matters they thought the nurses should handle themselves. In these situations, disagreement between doctor and nurses could arise. The intermediate position of being the doctor in charge and at the same time needing training and surveillance was experienced as tough. Still, they usually found it instructive to harvest from the nurses’ experience and knowledge. One of the participants expressed this mixed role:

*“To find the balance between having the responsibility as a doctor and listening to the nurses who have all the experience. When I didn’t know the patient, I had to lean on their evaluation and knowledge. I found it impressing when they could come to me and tell that death was imminent because they saw some indefinite signs – this smell, this glow in the eyes or… – I remember thinking that “OK, you’ve got experience I don’t have yet when it comes to evaluate those silent signals from the elderly.” I must lean on their judgement.” (Karen)*

## Discussion

In nursing homes the newly qualified doctors experienced that their calm presence at a deathbed could give patient, family and nurses relief and safeness. Challenging dialogues and multidisciplinary team-work taught them how to balance information and decisions, uncertainty and competence. Below, we discuss interpretations, strengths and limitations of these findings.

### What is known from before – what does this study add?

There is a growing interest for nursing homes as arena for medical education and interprofessional learning [23, 24]. The aging population is living with and dying from complex chronic conditions, including dementia. Education of doctors should mirror this changing demography and health related challenges. Many doctors find elderly care medicine less interesting than other clinical fields [25–27], geriatrics and nursing home medicine being ranged on the bottom of the medical status hierarchy [28]. Research in this particular clinical setting can contribute to enhanced interest and quality improvement, and also provide useful knowledge to medicine in general.

Previous studies have shown how nursing homes can offer medical students and newly qualified doctors a broad range of specific geriatric and long term care learning outcomes [12, 29, 30]. In this study we focused on EOL care and dialogues about death, emphasizing the impact of situated learning on development of doctors’ professional role. Our analysis revealed how the nursing home setting may give interns access to learning experiences through close interaction and dialogues with patients, relatives and nurses. The impact of internship in nursing homes varies with organisation and content. Previous research has demonstrated less supervision in nursing home practice than in hospital training, giving more autonomous learning experiences, but also loss of learning possibilities [29]. Our results indicate that quantity of nursing home practice and responsibility under supervision are important factors [31].

The influence on maturing of skills and attitudes through practicing as an inexperienced doctor in a multidisciplinary setting became obvious when we applied the theory of situated learning [15]. Our analysis emphasized how a learning community different from the hospital culture may support newly qualified doctors developing their professional identity. During the nursing home internship they evolved from being insecure concerning death and dying into competent doctors with modified attitudes to medical tasks and a good grip on EOL care. The theoretical perspectives made us aware not only of what the participants had learnt, but also in which ways this specific practice culture may promote new attitudes and skills. The concept “legitimate peripheral participation” drew our attention towards some of the difficulties and dilemmas the newly qualified doctors encountered [15]. For example, our analysis revealed the gradual development from insecurity to confidence in challenging dialogues about serious illness and end-of-life, guided by a combination of the nurses’ knowledge of patient and family and their own medical competence. The interdisciplinary setting in nursing homes gave the young doctors insight and experience in how their medical decisions were dependent on the skills of and communication with other professionals [32].

Nursing home patients and their relatives expect doctors to be involved and available in EOL care [8]. Medical skills and reflective attitudes towards death and dying are, however, often underdeveloped in doctors, who tend to avoid dialogues about existential questions [33, 34]. Systematic approach and education schemes can support the development of professional confidence towards death [35–37]. The traditional doctor’s role is often considered to be detached, objective and omnipotent. Adapting such a professional role can violate personal attitudes and values [38–40]. Our analysis demonstrated how some of the young doctors had positive experiences sharing their own insecurity and vulnerability during such conversations, similar to how general practitioners sometimes use their own vulnerability successfully in consultations [41].

EOL care affects not only patients, but also their families. The impact of patients’ personal history and circumstances often disappears focusing on diagnostic and therapeutic procedures. During internship in primary care this aspect can become more visible [5, 42]. Our findings demonstrate specifically how learning EOL care in nursing homes can provide newly graduated doctors with attitudes and skills which are useful in all clinical contexts, by offering experiences of personal and professional growth through close contact with family and patient. In medical education death is traditionally conveyed as defeat [43]. Our study revealed how interns perceived death being welcomed and relieving after a long life, and how they understood the relevance of relationships and life history for medical decisions. Such insight can be useful for doctors regardless of medical specialty by widening the doctors’ perspectives and their attitudes towards death. Nursing homes can thus provide a broader understanding of and approach to not only death and dying, but also confronting prevailing cultural standards of doctors’ action imperative and supreme resoluteness.

### Strengths and limitations

This study was conducted in one county in northern Norway with nursing homes located in rural as well as urban settings. Participants’ experiences from internship in nursing homes were rich and varied in content and organisation. Thus we argue that our findings may be applied in various nursing home settings, and the outcome of learning might also be transferable to other arenas where doctors encounter death and dying, such as hospital wards and home deaths.

The scope of this study was young doctors’ experiences, emphasizing the development of the medical practitioners’ role in this specific learning context. We did not interview their supervising doctors or nurses. We therefore cannot say how the community of practice assessed the interns’ performance and learning process.

Our research questions could have been explored by individual in-depth interviews focusing on participants’ individual learning experiences. Our choice of focus groups as interview setting gave participants the opportunity to inspire each other in sharing their nursing home experiences. The focus group method provided a flexible and reflexive setting for this conversation. The group setting might possibly have hampered openness. Participants knew each other from the coaching assemblies. This provided them with a safe arena, but could also be a hindrance for sharing difficult experiences. Our impression was that the participants openly shared both good and bad situations.

The interviewer (AF) is a nursing home doctor in the same county in which the participants completed their internships, with long experience from EOL care in nursing homes. This local and professional background provided a solid base for the inquiry and the role as moderator, but could also prone for posing leading questions and inflict on analysis and interpretation, for example by exaggerating the value of nursing homes as learning arena. Nevertheless we considered the nursing home background and extensive field knowledge as an advantage in our setting. During analysis we deliberately focused on the positive learning experiences, realizing that we thereby put less emphasis on the impediments for using this learning arena. Analysis confirmed our preconceptions that nursing homes can give surplus insight and training in difficult dialogues about death and dying. Another preconception was that long distance to hospital could be an important source of concern for the newly qualified doctors. To our surprise, distance was not one of the main problems for the participants. Their preoccupation regarded relationship with patients, families and nurses, as well as their own initial insecurity in difficult dialogues.

Discussing death and dying involves existential and psychological reflections. These elements were part of the discussions, but we did not explore them explicitly due to our focus on learning experiences.

## Conclusions

There is a considerable potential for training doctors in EOL care in nursing homes, which can be developed and integrated in medical education. This practice based learning arena offers newly qualified doctors close interaction with patients, relatives and nurses, teaching them to perform difficult dialogues, individualize medical decisions and balance their professional role in an interdisciplinary setting.

## Abbreviation

EOL Care: End of life care
